# Applicability of machine learning algorithm to predict the therapeutic intervention success in Brazilian smokers

**DOI:** 10.1371/journal.pone.0295970

**Published:** 2024-03-04

**Authors:** Miyoko Massago, Mamoru Massago, Pedro Henrique Iora, Sanderland José Tavares Gurgel, Celso Ivam Conegero, Idalina Diair Regla Carolino, Maria Muzanila Mushi, Giane Aparecida Chaves Forato, João Vitor Perez de Souza, Thiago Augusto Hernandes Rocha, Samile Bonfim, Catherine Ann Staton, Oscar Kenji Nihei, João Ricardo Nickenig Vissoci, Luciano de Andrade

**Affiliations:** 1 PhD Student in the Postgraduate Program in Health Sciences, State University of Maringa, Maringa, Parana, Brazil; 2 Master in Computer Sciences, State University of Maringa, Maringa, Parana, Brazil; 3 Professor in the Morphological Sciences Department, State University of Maringa, Maringa, Parana, Brazil; 4 Professor in the Department of Medicine, State University of Maringa, Maringa, Parana, Brazil; 5 Global Emergency Medicine Innovation and Implementation Research Center, Duke University School of Medicine, Duke Global Health Institute, Durham, North Carolina, United States of America; 6 Master Student in the Postgraduate Program in Health Sciences, State University of Maringa, Maringa, Parana, Brazil; 7 Assistant Professor of Emergency Medicine and Global Health, Duke Global Health Institute, Department of Emergency Medicine, Duke University School of Medicine, Durham, North Carolina, United States of America; 8 Professor in the Center of Education, Literature and Health, Western Parana State University, Foz do Iguaçu, Parana, Brazil; 9 Professor in the Postgraduate Program in Health Sciences, State University of Maringa, Maringa, Parana, Brazil; UFSJ: Universidade Federal de Sao Joao del-Rei, BRAZIL

## Abstract

Smoking cessation is an important public health policy worldwide. However, as far as we know, there is a lack of screening of variables related to the success of therapeutic intervention (STI) in Brazilian smokers by machine learning (ML) algorithms. To address this gap in the literature, we evaluated the ability of eight ML algorithms to correctly predict the STI in Brazilian smokers who were treated at a smoking cessation program in Brazil between 2006 and 2017. The dataset was composed of 12 variables and the efficacies of the algorithms were measured by accuracy, sensitivity, specificity, positive predictive value (PPV) and area under the receiver operating characteristic curve. We plotted a decision tree flowchart and also measured the odds ratio (OR) between each independent variable and the outcome, and the importance of the variable for the best model based on PPV. The mean global values for the metrics described above were, respectively, 0.675±0.028, 0.803±0.078, 0.485±0.146, 0.705±0.035 and 0.680±0.033. Supporting vector machines performed the best algorithm with a PPV of 0.726±0.031. Smoking cessation drug use was the roof of decision tree with OR of 4.42 and importance of variable of 100.00. Increase in the number of relapses also promoted a positive outcome, while higher consumption of cigarettes resulted in the opposite. In summary, the best model predicted 72.6% of positive outcomes correctly. Smoking cessation drug use and higher number of relapses contributed to quit smoking, while higher consumption of cigarettes showed the opposite effect. There are important strategies to reduce the number of smokers and increase STI by increasing services and drug treatment for smokers.

## Introduction

The World Health Organization estimates that by 2023, for each US$1 invested in prevention and control of noncommunicable diseases, there might be a return of at least US$7. These strategies can also avoid 15% of premature deaths and save around 8.2 million lives in low- and middle-income countries [1]. However, around 1.3 billion people worldwide (22.3% of the global population) smoke regularly, despite the development of numerous strategies to control tobacco consumption and as a result, more than eight million people die every single year [2, 3]. This makes smoking the leading cause of illness, poverty, and death worldwide, and one the most important global threats [2, 3].

Tobacco control is a part of the SimSmoke program, a widely adopted strategy worldwide and one of the main goals of which is smoking cessation [4–7]. Smoking cessation is also an important public health policy particularly in low- and middle-income countries and in geographical regions where around 80% of tobacco consumers live and negative effects of this habit are more evident [2, 3].

In Brazil, a middle-income country in South America, smoking cessation intervention centers, which follows the Brazilian Ministry of Health (BMH) smoking cessation guideline [8], attended around 800,000 Brazilians between 2005 and 2014 [3, 9]. However, in 2019, around 12% of people over 18 years old were still smokers in Brazil [10].

Programs to treat smokers reduced their number by approximately 7% and increased smoking cessation rate by 55% between 1989 and 2010 [4]. In view of the foregoing, the effectiveness of treatment could potentially be improved by identifying patients who are more likely to succeed if they attempt smoking cessation programs [2, 3]. However, developing a new method that increases the participation and permanence in the treatment, and consequently the therapeutic success, is still a challenge especially in low- and middle-income countries. In addition, treating smokers in Brazil is costly [11] and has been losing its efficacy over time [12].

Machine learning (ML) can be a key resource to improve the therapeutic success of smokers, since it gets together the variables with specific characteristics, such as the treatment effects, level of nicotine dependence, and so on, which individually can contribute to the defined outcome. The combination of them gives us a diagnostic or prognostic of health problems [13–15], and it can be used to better comprehend the dataset structure associated with smoking quit and to construct user-friendly tools, such as risk equators [16–19].

Previous studies show that for smokers, ML application showed good results in the empirical study of smoking cessation intervention in Korea (precision between 67.3% and 87.7%) [15], and nicotine dependence evaluation in Jordanian women (precision of 82.0%) [13]. However, in Brazilian healthcare services, the ML application for screening of patients is scarce and as far as we know it was used only in the cardiovascular risk evaluation [18], highlighting lack of screening of variables related to therapeutic intervention success (TIS) in Brazilian smokers using ML algorithms. Our study aims to evaluate the applicability of classificatory supervised ML algorithms in predicting correctly the TIS in Brazilian smokers and identifying intrinsic characteristics that increase the probability of smoking cessation.

## Material and methods

### Study design

We conducted a retrospective, observational, cross-sectional, and descriptive study using machine learning tools to predict therapeutic intervention success (TIS) in Brazilian smokers, based on its application in medicine [20] and the Transparent Reporting Multivariable Prediction Model for Individual Prognosis and Diagnosis (TRIPOD) guideline [21].

### Data sources

Our dataset was composed of secondary data of patients who participated in the smoking cessation program called “Extension Project of Treatment and Care to Tobacco Users in the population of the municipality of Maringa and Region” (free translation), carried out from January1^st^, 2006 to December 31^st^, 2017, which evaluated the response of the participants to this program. These data were collected from January 1^st^, 2020 to June 30rd, 2021 and, accessed and analyzed from July 1^st^, 2021 to May, 2023.

The program referred above was carried out at the State University of Maringa, Parana, Brazil, and followed the Brazilian Ministry of Health (BMH) smoking cessation guideline [8]. Intervention was coordinated by two healthcare professionals, and it was offered, in general, in groups of 10 to 15 people, and gave to the participants information about the harmful effects of smoking, the benefits of quitting and support to reduce nicotine dependence [22].

For this, all smokers had their sociodemographic, clinical and smoking profile data collected to structure the medical records. Smokers were divided into groups and underwent cognitive-behavioral therapy, consisting of seven sessions (four structured according to the BMH protocol and three supporting sessions), one session per week [8, 23].

The BMH protocol also states that smokers who participate in cognitive-behavioral treatment can receive drug therapy (bupropion hydrochloride and/or nicotine replacement drugs) if necessary [8, 23]. In the analyzed program, smokers who did not quit smoking during the cognitive-behavioral therapy were allowed to participate in the next group as many times as necessary.

### Outcome

The outcome selected for this study was smoking cessation during the period of 42 days (7 meetings) of the cognitive-behavioral treatment, to measure how the models are trustworthy to identify the participants who will quit smoking if they received such treatment.

### Data selection

#### Selection of the participants

**Fig 1:**
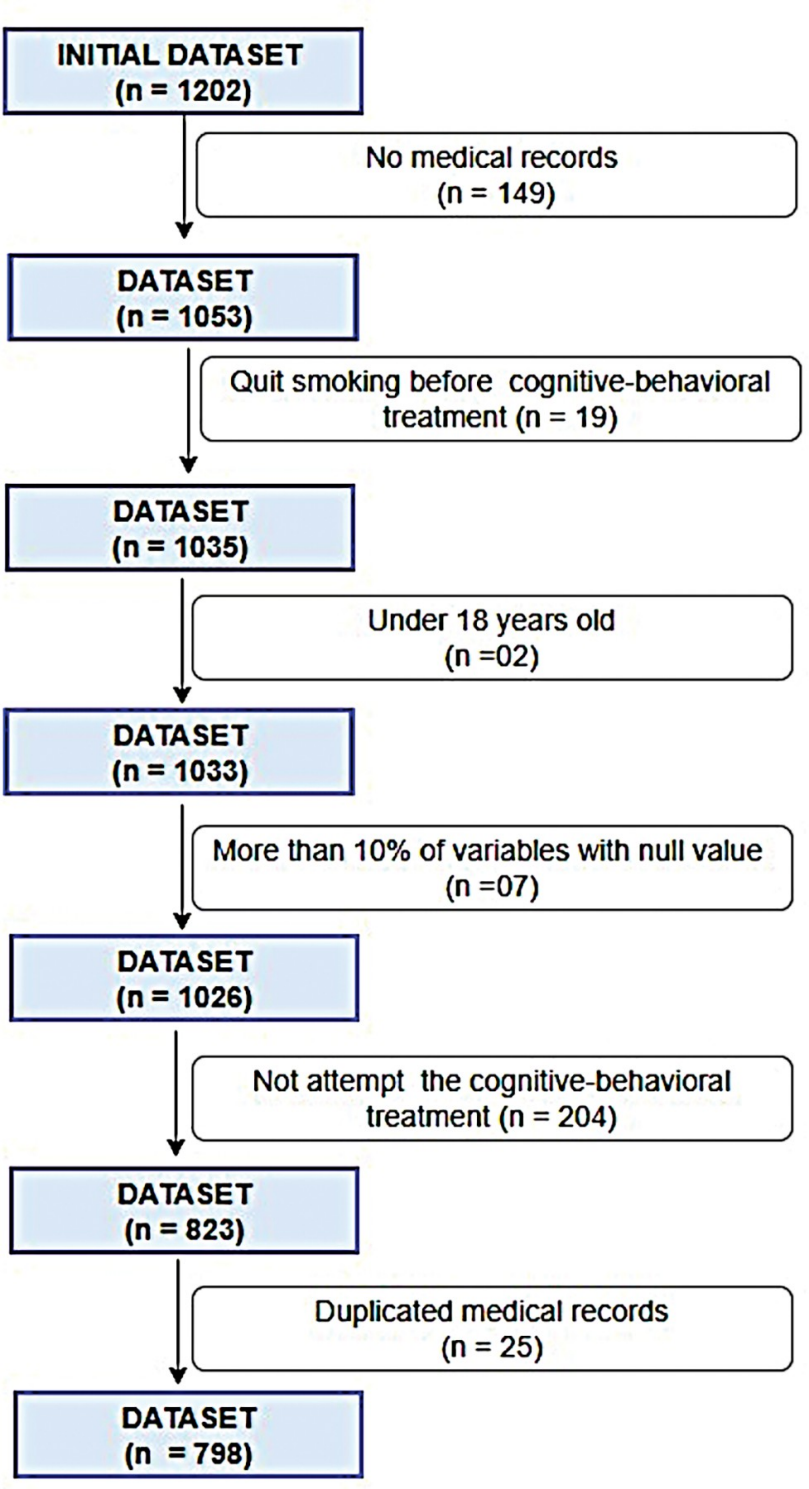
Flowchart of medical records selected for descriptive and predictive analysis.

The database for this study was composed by smoking individuals. Intervention characteristics evaluated routinely in this smokers’ treatment program were from 1202 smokers registered in 80 treatment groups between 2006 and 2017. We excluded 381 smokers who had no medical records, quit smoking before the cognitive-behavioral treatment, were under 18 years old, had more than 30% of variables with null answers in the medical records, and/or did not take part in the cognitive-behavioral treatment. There were 25 duplicated medical records and in these cases only the last group which the patient took part in was considered. The remaining 798 medical records were used for descriptive and predictive analysis (**Fig 1**).

#### Selection of variables

Among 42 variables available (see complementary materials), we excluded those that showed more than 10% of null data, the response was very ample (i.e.: jobs), more than one possible answer per patient (i.e.: reasons to become smokers or who encourage smoking), variables linked together or those linked with smoking cessation in Pearson’s correlation test (more than 70% correlation) [24] (Table 1).

**Table 1:** Variables selected for this study.

Variables	Explanation	Classification	Possible answers
Quit	Smoking cessation during the cognitive-behavioral treatment	Binominal	No = 350
			Yes = 448
Sex	Sex of the patient	Character	Male = 372
			Female = 426
Marital status	Marital status of the patient	Character	Single = 373
			Couple = 414
			Not available = 011
Educational level	Evaluate the educational level of patients (in years of study)	Character	Zero = 008
			1-8 = 204
			9-11 = 315
			12 or more = 264
			Not available* = 007
Self-reported disease	Self-report of disease, besides the smoking during the medical records structuration	Binominal	No = 323
			Yes = 4 74
			Not available = 001
Smoking cessation drug	Use of drugs to help in the smoking cessation during the cognitive-behavioral treatment	Binominal	No = 294
			Yes = 504
Tobacco consumption time	Time of tobacco consumption (in years)	Range of number	1 to 10 = 062
			11 to 20 = 122
			21 to 30 = 201
			31 to 40 = 237
			41 to 50 = 134
			More than 50 = 035
			Not available = 007
Number of cigarettes	Number of cigarettes took per day	Range of number	1 to 10 = 159
			11 to 20 = 431
			21 to 30 = 114
			31 to 40 = 074
			More than 40 = 18
			Not available = 002
Nicotine dependence level	Nicotine dependence level according to Fagerstrom test**	Character	Very low = 098
			Low = 170
			Middle = 156
			High = 269
			Very high = 105
Relapse	Number of relapses of smoking cessation before taking part in the cognitive-behavioral treatment	Character	0 = 168
			1 = 525
			2 = 057
			3 = 023
			More than 3 = 025
Quit attempts	Number of smoking cessations attempts before taking part in the cognitive-behavioral treatment	Character	0 = 098
			1 = 237
			2 = 128
			3 = 091
			More than 3 = 244
Motivation	Evaluate if the patient was motivated to quit smoking	Binominal	No = 075
			Yes = 723

*Number of patients that did not answer the question.

**Test model is available at: https://www.heartonline.org.au/media/DRL/Fagerstrom_test_of_nicotine_dependence.pdf.

#### Associative analysis

Odds ratio was calculated for each variable selected above in order to evaluate the association between each answer and the outcome with 95% confidence interval by adopting the first option as a reference [25]. Final results were plotted as a forest plot [26]. The analysis was performed using R Studio software.

#### Predictive model development

**Fig 2:**
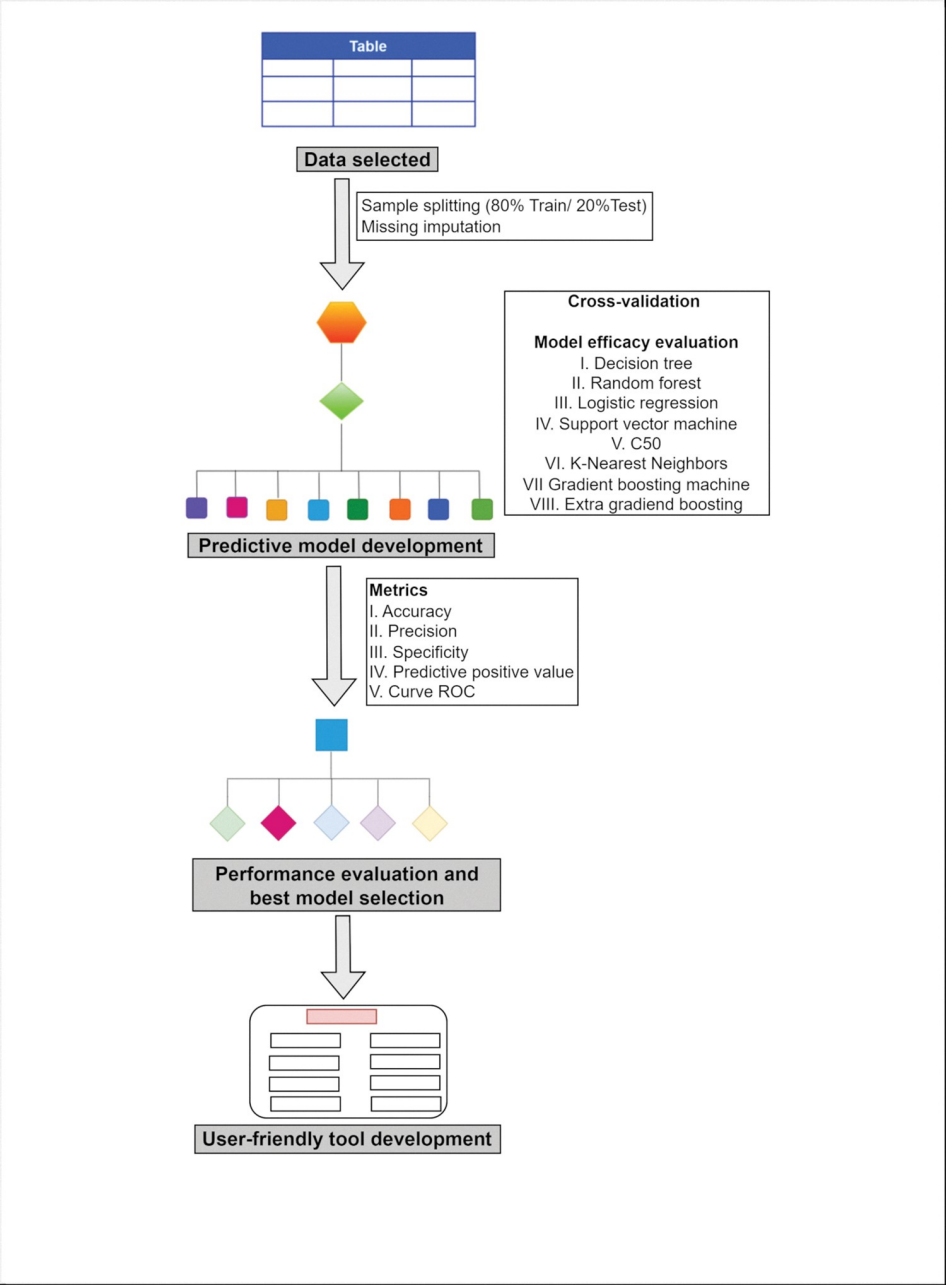
Flowchart of supervised and classificatory machine learning algorithms performance evaluation and best model selection.

We tested a classificatory supervised ML algorithms performance to evaluate TSI in Brazilian smokers (**Fig 2)**.

*Data splitting and missing imputation*. To standardize the classificatory supervised machine learning algorithms by *RStudio* software for each group, the dataset was randomly split into 80% of data for training ML algorithms, and 20% for testing by using the *caret* package [27]. For each algorithm and group, missing values were imputed 10 times using the *mice* package (multivariate imputation chained equation) [28].

*Cross-validation*. During model performance evaluation of each algorithm and group, we performed cross-validation 10 times for training and testing groups. In both groups, the dataset was split into 10 parts. Nine parts were used for internal training and the other used for validation [29].

*Algorithms discrimination*. Eight ML algorithms based on the CARET derived models were used in order to evaluate their ability to correctly predict TIS in smokers. We tested 4 tree-base algorithms: Decision Tree (DT) [30], Random Forest (RF) [31], C5.0 [32], and Gradient Boosting Machine (GBM) [33]. We also tested 4 no tree-based algorithms: Logistic Regression (LR) [34], K-nearest Neighbors (KNN) [35], Support Vector Machine (SVM) [36], and Extra Gradient Boosting (XGB) [37].

*Decision tree flowchart*. To visualize how variables combine each other to map a possible outcome, we plotted a decision tree flowchart [38].

*Algorithms performance evaluation*. In order to reduce outlier effects in the final decision of each algorithm, we calculated the mean values and standard-deviation (m±SD) of the following metrics: accuracy (ACCU), sensitivity (SENS), specificity (SPE), predictive positive value (PPV) and area under the receiver operating characteristic curve (AUC) [14, 39–41].

*Most important algorithms selected and user-friendly tool development*. The most effective ML algorithm was chosen based on the mean value of PPV, which measures the probability of true positive results among all samples predicted as positive [40, 41]. This algorithm was used to calculate the highest value of variable’s importance [42] and construct the therapeutic intervention success probability equator (TS-equator) [16]. The TS-calculator is a computer-linked tool where healthcare professionals can input data and the software calculates the probability of a patient quitting smoking during cognitive-behavioral treatment.

#### Ethical aspects

This study was approved by the Permanent Ethical Committee in Research involving Humans of State University of Maringa (UEM) under the protocol number: 468,857/202 and Resolution number 466/2016 of Brazilian Ministry of Health who allowed us to work with secondary data without informed consent forms.

## Results

### Participants characteristics

The participants evaluated in our study had an average age of 46±2.3 years and were mostly female (53.3%), married (51.9%), with at least 9 years of education (72.6%) and consumed 11 to 20 cigarettes per day (73.9%). Regarding clinical aspects, the majority had associated comorbidities (59.4%) and used anti-smoking medications (63.2%).

It was also observed that 73.2% had medium to very high dependence on nicotine, 78.9% had at least one relapse before screening, 87.7% had made at least one attempt of quitting before seeking an anti-smoking treatment center and 90.6% were motivated to stop smoking.

Analyzing only the 448 participants who stopped smoking (56.1%), it was observed that the average time without smoking was 26.9±9.2 days, considering only the 42 days of monitoring by the extension project, indicating that on average the participants quit smoking at the 16^th^ day of treatment and did not relapse for the rest of the period. Furthermore, five of these participants had previously participated in the treatment at the same smoking cessation intervention center.

### Associative analysis

**Fig 3:**
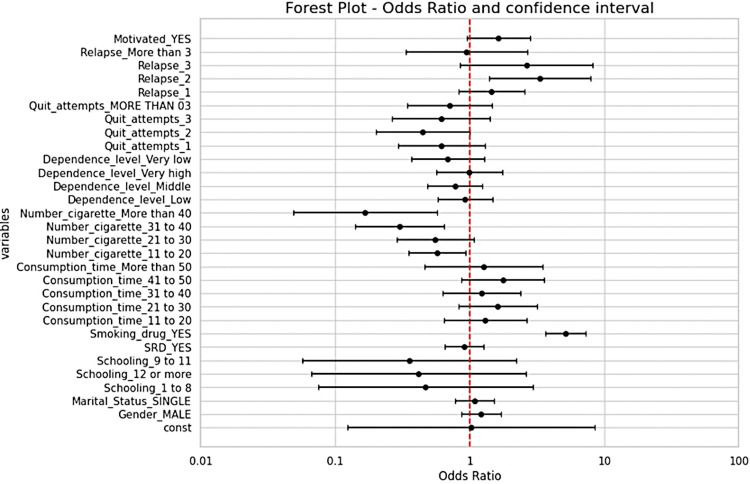
Forest plot of odds ratio and confidence interval of variables according to smoking cessation.

Use of drugs to help quitting smoking and increase of the number of relapses also promoted a positive outcome related with therapeutic intervention success (TIS). On the other hand, higher consumption of cigarettes resulted in a negative outcome. The use of drugs to help in smoking cessation also showed the highest OR (4.42 [3.25-6.00]) (**Fig 3**).

### Decision tree flowchart

**Fig 4:**
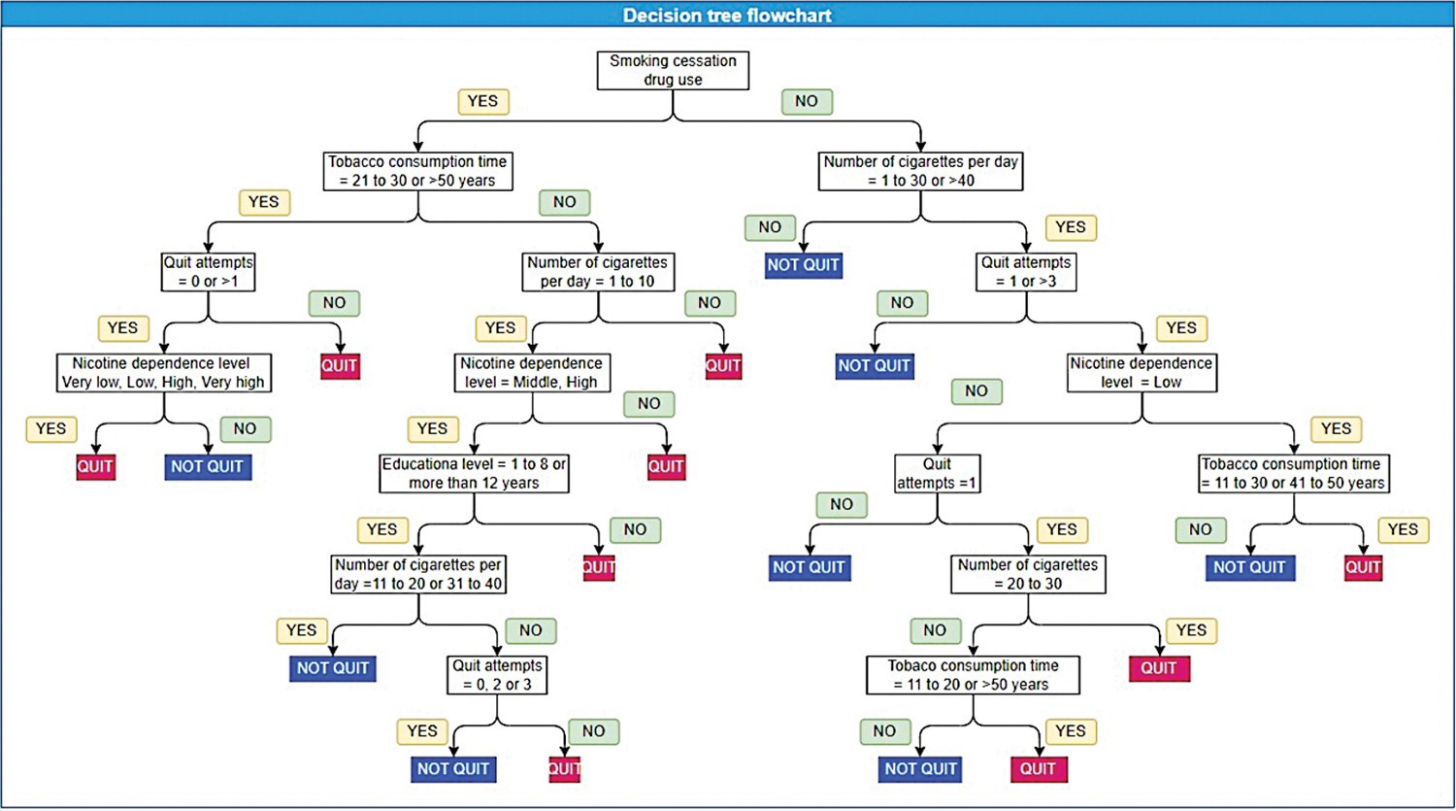
Decision tree flowchart.

The decision tree flowchart was built to show hierarchy of variables, splitting it in two groups according to the answer of that variable, until the outcome (quit or not quit) is achieved, the closer a variable is to the roof of the decision tree, the more important it is in the model’s decision-making process. This flowchart shows us that the average number of branches was 5.12 ± 1.87 and the use of drugs to help with smoking cessation is the roof of the tree, so this variable was the most important to smoking cessation (**Fig 4**).

### Evaluation of machine learning algorithms performance

The mean global values were: 0.675 ± 0.028 for accuracy, 0.803 ± 0.078 for sensitivity, 0.485 ± 0.146 for specificity, 0.705 ± 0.035 for PPV, and 0.680 ± 0.033 for AUC. After analyzing each algorithm individually, we noticed that the accuracy, specificity and PPV were higher in SVM while sensitivity was higher in KNN **(Table 2)**. As the main objective of this study was to find the smokers who has high probability of quit smoking during the 7 weeks of the treatment offered by the healthcare center, we chose SVM as the best model based on the mean value of PPV, since it measures the probability of true positive results among all samples predicted as positive.

**Table 2:** Mean and standard deviation of the predictive values of machine learning models evaluated.

	Accuracy	Sensitivity	Specificity	PPV*	AUC**
**Decision Tree**	0.682±0.020	0.771±0.029	0.548±0.026	0.719±0.014	0.658±0.019
**Random Forest**	0.674±0.020	0.785±0.036	0.506±0.022	0.705±0.010	0.655±0.009
**Logistic Regression**	0.681±0.044	0.824±0.093	0.466±0.246	0.709±0.058	0.686±0.066
**SVM**	0.688±0.029	0.775±0.051	0.556±0.098	0.726±0.031	0.703±0.019
**C5.0**	0.651±0.018	0.726±0.032	0.538±0.068	0.703±0.023	0.694±0.026
**K-Nearest Neighbors**	0.683±0.018	0.924±0.039	0.320±0.085	0.672±0.020	0.703±0.013
**GBM** ***	0.668±0.037	0.848±0.087	0.397±0.212	0.686±0.046	0.664±0.017
**XGB** ****	0.679±0.019	0.768±0.021	0.545±0.032	0.717±0.0016	0.681±0.019

*Predictive positive value

**Area under the receiver operating characteristic curve

***Gradient boosting machine

**** Extra Gradient Boosting.

The importance of variables for the best model (SVM) were: 100.00 for use of drugs to help in the smoking cessation, 18.6 for motivation to quit smoking, 15.3 for number of relapses, 7.8 for number of cigarettes consumed per day, 7.8 for marital status, 7.5 for number of quit attempts, 5.5 for tobacco consumption time, 4.5 for nicotine dependence level, 4.3 for educational level and 3.6 for self-reported disease.

**Fig 5:**
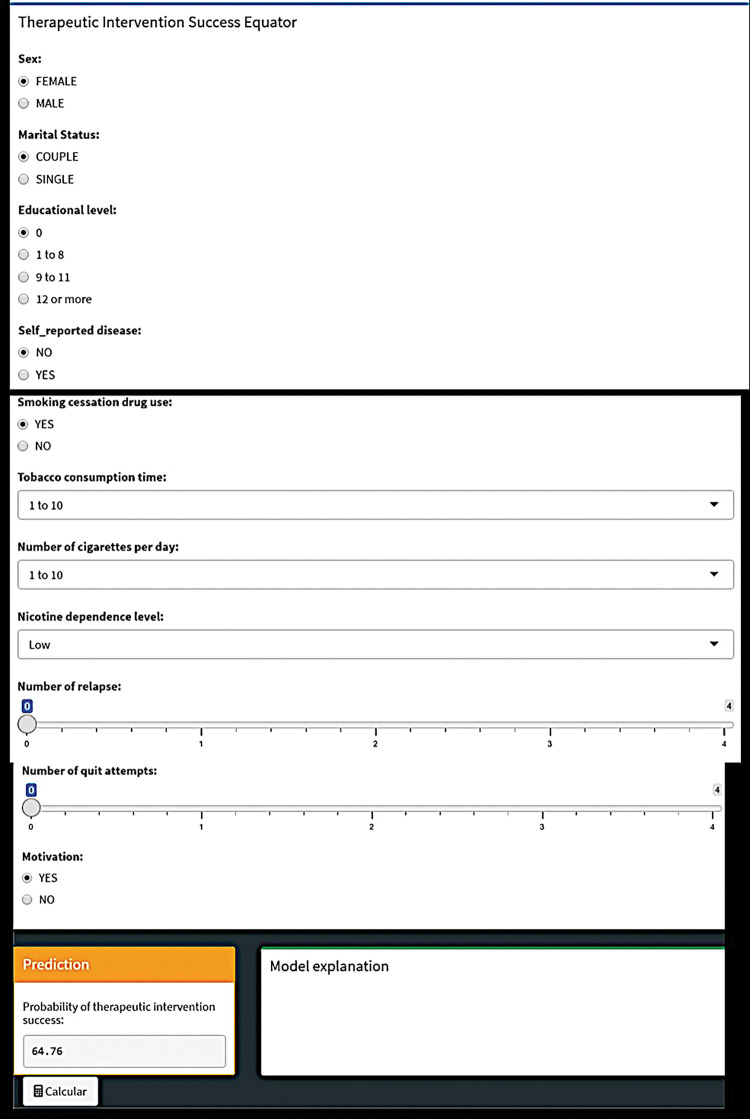
Image of therapeutic intervention success probability equator prototype.

Finally, a prototype of therapeutic intervention success probability equator (TS-equator) was developed as we can see in Fig 5.

## Discussion

Prevention and control of noncommunicable diseases can be a key to saving money and lives, especially in low- and middle-income countries [1]. However, increasing efficacy of smoking cessation intervention is a difficult challenge worldwide. In order to aim in the management and draw strategies to solve this global threat, we evaluated the applicability of eight ML algorithms in predicting TIS in smokers and influence of variables in the efficacy of smoking cessation program in Brazil.

We noticed that, except for KNN and GBM, all models tested showed a PPV higher than 70%, highlighting their potential to be used by smoking cessation programs, and among these, the SVM was the best model. Since PPV is the ability to detect true positive results among all samples predicted as positive [14, 42], in our study it measures how the model is trustworthy to identify the participants who will quit smoking if they received the cognitive-behavioral treatment. So, it can be used to establish priorities when the demand is higher than the capacity of the program and try to find new methodologies for people who have difficulty to achieve therapeutic success. However, it should not replace screening and initial approach by healthcare professionals.

Support vector machine is the most tested ML algorithm worldwide to solve public health problems [40]. The PPV obtained by the best model (SVM) in our study (0.726) is higher than that for severe dengue infection prognostic in Thailand using logistic regression model (PPV= 0.687) [43]. According to the literature, this algorithm, especially the non-linear type, is also useful to make predictions with small amounts of data, due to its mechanism of analysis (subset of training points) [44].

Previous study also describes that PPV can be increased combining different parameters [45]. Aiming to understand how predictive variables work, we performed complementary analysis which showed that use of drugs that help quitting smoking presented higher OR with therapeutic intervention success. It was also the most important variable and roof of the decision tree, indicating that it surpasses other sociodemographic and clinical profiles to predict the outcome of patients participating in the smoking cessation program. A previous study in Brazil had already demonstrated that drug therapy was directly related to smoking cessation [23, 46], even the Brazilian Ministry of Health recommends the use of bupropion hydrochloride and nicotine replacement drugs in heavy smokers treated by healthcare centers [8].

The high association between smoking cessation and drug therapy may be due to the reduction of signs and symptoms of nicotine withdrawal, such as anxiety, aggressiveness, difficulty in interpersonal interactions, among others [47]. Despite this, use of these compounds should be performed under doctor’s supervision, as they can interact with beta-blockers, antidepressants, and antipsychotics, altering their effectiveness and contributing to the occurrence of drug interactions and increasing the risks of side-effects, such as hypotension, hypertension, so on [48–50].

Another variable directly associated with smoking cessation was the number of relapses before taking part in the behavioral-cognitive treatment. Thus, consolidation of the decision to quit smoking, can be measured mainly by the number of previous attempts and relapses. In this context, BMH states that, in general, the smokers make five attempts before achieving success, and in patients undergoing treatment, the identification of factors that led to relapse helps health professionals to deal with skills that allowed smoking cessation in previous times and avoid new relapses [8, 22].

On the other hand, higher consumption of cigarettes per day showed the opposite effect and similar results were found in patients treated by a healthcare center in the city of Belém, Pará, Brazil [51] and Joinville, Santa Catarina, Brazil [52]. However, how the number of cigarette consumption influences smoking cessation is still not understood in the literature.

Previous research has consistently shown that motivation level plays a pivotal role in the frequency of previous quit attempts and relapses, even though attempting to quit does not guarantee to achieve the goal [53, 54].

It is also known that therapeutic success is time dependent, and some studies describe that the probability of relapses increased with the time analyzed [55, 56]. However, anamnesis after group meetings is made difficult by the high number of patient losses, therefore, in the present study a time period of 7 weeks was adopted to determine which patients would be most likely to quit smoking.

Our results indicate that after refinements in the ML algorithms to increase their efficacies and conversion of the TIS into widespread user-friendly tools, the healthcare managers and professionals, researchers and patients can benefit from it in different ways.

Smoking cessation centers can optimize resource allocation and thus personalize treatment approaches, identifying early smokers with higher probability of therapeutic success and providing a more intensive intervention for those with a lower probability of TIS. Health managers can use the insights from the study to shape public policies and treatment guidelines, making the resources (e.g., medication) more accessible. The pharmaceutical factory can be informed about which medication or therapies are more effective, guiding research and development, as well as marketing strategies, thereby renewing the incentive to develop or improve smoking cessation drugs.

The results of this study can serve as a basis for public education campaigns about the factors contributing to smoking cessation success. Health application developers can incorporate the TS equator into smoking cessation applications to provide personalized feedback to users, encouraging them to stick with their cessation goals. This study provides a model and approach that other researchers can replicate or adapt in different populations or settings, thus expanding the knowledge about smoking cessation and machine learning techniques.

So, using ML-based calculator to predict TIS in Brazilian smokers can be a valuable tool to complement traditional intervention approaches, since it works in the same way that the risk calculator developed to predict lesions in patients with traumatic brain injuries [16, 19], diabetes [17] and cardiovascular diseases [18], even the metric evaluated in these calculators are the, this means, the ability to identify correctly the positive sample. By getting together quantitative insights from the tool with a deep understanding of psychosocial, cultural and environmental factors, healthcare professionals can create more effective and personalized approaches to helping individuals quit smoking. However, it is essential to recognize and address the implications of its use in clinical practice. One of the main potential risks is that healthcare professionals may overly rely on the results of this tool, leading to possible biases in decision-making. If an individual receives a low score, this may discourage both the patient and healthcare provider from seeking and implementing smoking cessation interventions.

This is particularly concerning as an individual’s determination and motivation to quit smoking may not be fully captured by the tool, especially when considering that many psychosocial factors, cultural and environmental factors also play a crucial role in the process of quitting smoking. Healthcare professionals should be trained to use this tool as one of several assessment tools and not as a definitive determinant of a patient’s ability to quit smoking. Thus, complementing the tool score with a comprehensive assessment of the patient’s situation, including their motivation, social support, perceived barriers, and other relevant factors that may influence their journey to smoking cessation.

Moreover, some limitations related to the use of retrospective secondary data still remain in our study. First, the limited number of patients analyzed, possible failures to collect data for registration and the use data from single smokers’ treatment program can lead to a restricted scenario and difficulty in applying the therapeutic intervention success probability equator (TS-equator) in other regions. Therefore, even though computer-based techniques are an important way to work with clinical datasets, it is important to evaluate and validate these algorithms using data from other smoking cessation programs.

In the present study, all models showed a specificity of 50% or lower demonstrating their low capacity to correctly find the participants who will not quit smoking during the cognitive-behavioral treatment, resulting in high probability of false positives [57, 58]. This limitation in model specificity is relevant to the analysis as it is intrinsically related to its ability to correctly distinguish negative cases from false positives, making the clinical decision-making difficult. It also may result in a high number of participants who will receive the treatment without achieving the goal and it can even be used to reduce the manual validation [58].

In general, tobacco consumption is a multifactorial event, even the BMH describes very little data collected by the patients assisted by a healthcare center. Sociodemographic, clinical, smoking profile, and ex-smokers’ outcome after treatment should be evaluated to reduce the probability of false negatives results and strengthen this kind of investigation.

## Conclusion

The SVM was the best model, predicting the higher percentage of patients quitting smoking if they receive the cognitive-behavioral treatment, demonstrating its high ability to be used in the real-world to establish priorities when the demand is higher than the capacity of the program.

Moreover, the use of smoking cessation drugs and occurrence of more relapses before taking part in the cognitive-behavioral treatment have contributed to quitting smoking, suggesting that increase of healthcare accessibility and drug therapy may be a key to reduce the number of smokers.

## References

[1] World Health Organization. Noncommunicable diseases country profiles; 2018 [cited 2023 June 21]. Database: WHO [Internet]. Available from: https://apps.who.int/iris/handle/10665/274512.

[2] World Health Organization. Tobacco; 2022 [cited 2023 June 21]. Database: WHO [Internet]. Available from: https://www.who.int/news-room/fact-sheets/detail/tobacco.

[3] Pan-American Health Organization. Tabaco; 2023 [cited 2023 Jun 21]. Database: PAHO [Internet]. Available from: https://www.paho.org/pt/topicos/tabaco.

[4] Levy D, Almeida LM, Szylo A. The Brazilian SimSmoke policy simulation model: the effect of strong tobacco control policies on smoking prevalence and smoking-attributable deaths in a middle-income nation. PLoS Med. 2012; 9(11): e1001336. doi: 10.1371/journal.pmed.1001336 23139643 PMC3491001

[5] Sánchez-Romero LM, Yuan E, Ki Y, Levy DT. The Kentucky SimSmoke tobacco control policy model of smokeless tobacco and cigarette use. Int J Health Policy Manag. 2022; 11(5): 592–609. doi: 10.34172/ijhpm.2020.187 33131221 PMC9309926

[6] Maslennikova GY, Oganov RG, Boytsov S, Ross H., Huang A, Near A, et al. Russia SimSmoke: the long-term effects of tobacco control policies on smoking prevalence and smoking-attributable deaths in Russia. Tob Control. 2014; 23: 484–490. doi: 10.1136/tobaccocontrol-2013-051011 23853252 PMC4499848

[7] Levy D, Rodriguez-Buno RL, Hu T-w, Moran AE. The potential effects of tobacco control in China: projections from the China SimSmoke simulation model. BMJ. 2014; 348: g1134. doi: 10.1136/bmj.g1134 24550245 PMC3928439

[8] Ministério da Saúde. Portaria n° 761, de 21 de junho de 2016. Valida as orientações técnicas do tratamento do tabagismo constantes no Protocolo Clínico e Diretrizes Terapêuticas – Dependência à Nicotina; 2016 [cited 2023 Jun 21]. Database: MS [Internet]. Available from: https://bvsms.saude.gov.br/bvs/saudelegis/sas/2016/prt0761_21_06_2016.html.

[9] Portes LH, Machado CV, Turci SRB, Figueiredo VC, Cavalcante TM, Silva VLC. A política de controle do tabaco no Brasil: um balanço de 30 anos. Ciênc Saúde Colet. 2018; 23(6): 1837–1848. doi: 10.1590/1413-81232018236.05202018 29972492

[10] Brasil. Ministério da Saúde. Instituto Brasileiro de Geografia e Estatística. Pesquisa Nacional de Saúde: 2019 - Percepção do estado de saúde, estilos de vida, doenças crônicas e saúde bucal. IBGE, 2020. 113 p.

[11] Mendes ACR, Toscano CM, Barcellos RMS, Ribeiro ALP, Ritzel JB, Cunha VS, et al. Cost of the Smoking Cessation Program in Brazil. Rev Saude Publica. 2016; 50: 1–12. doi: 10.1590/S1518-8787.2016050006303 27849293 PMC5117528

[12] Pires GAR, Charlô PB, Marques FRDM, Covre ER, Paiano M, Salci MA. Análise do programa de controle do tabagismo em um município de médio porte do Paraná. Saude Colet. 2021; 11(67): 6789–6800. doi: 10.36489/saudecoletiva.2021v11i67p6789-6800

[13] Kharabsheh M, Meqdadi O, Alabed M, Veeranki S, Abbadi A. Alzyoud S A machine learning approach for predicting nicotine dependence. Int J Adv Comput Sci Appl. 2019; 10(3): 179–184. doi: 10.14569/IJACSA.2019.0100323

[14] Coughlin LN, Tegge AN, Sheffer CE, Bickel W. A machine-learning approach to predicting smoking cessation treatment outcomes. Nicotine Tob Res. 2020; 22(3): 415–422. doi: 10.1093/ntr/nty259 30508122 PMC7297111

[15] Davagdorj K, Lee JS, Pham VH, Ryu KH. A comparative analysis of machine learning methods for class imbalance in a smoking cessation intervention. Appl Sci. 2020; 10(9): 3307. doi: 10.3390/app10093307

[16] Rocha TAH, Elahi C, Silva NC, Sakita FM, Fuller A, Mnbaga B, et al. A traumatic brain injury prognostic model to support in-hospital triage in a low-income country: a machine learning-based approach. J Neurosurg. 2022; 132(6): 1961–1969. doi: 10.3171/2019.2.JNS182098 31075779

[17] Oliveira AR, Roesler V, Iochpe C, Schmidt MI, Vifo A, Barreto SM, et al. Comparison of machine-learning algorithms to build a predictive model for detecting undiagnosed diabetes – ELSA-Brasil: accuracy study. Sao Paulo Med J. 2017; 135(3): 234–46. doi: 10.1590/1516-3180.2016.0309010217 28746659 PMC10019841

[18] Ministério da Saúde. Aplicativo calcula risco de morte por doenças cardiovasculares; 2015 [cited 2023 Nov 06]. Database: MS [Internet]. Available from:https://www.unasus.gov.br/noticia/aplicativo-calcula-risco-de-morte-por-doencas-cardiovasculares.

[19] Terabe ML, Massago M, Iora PH, Rocha TAH, Souza JVP, Huo L, et al. Applicability of machine learning technique in the screening of patients with mild traumatic brain injury. PLoS One. 2023; 18(8): e0290721. doi: 10.1371/journal.pone.0290721 37616279 PMC10449130

[20] Verma AA, Murray J, Greiner R, Cohen JP, Shojania KG, Ghassemi M, et al. Implementing machine learning in medicine. CMAJ. 2021: 193(34): E1351:E1357. doi: 10.1503/cmaj.202434 35213323 PMC8432320

[21] Collins GS, Reitsma JB, Altman DG, Moons KGM. Transparent reporting of multivariable prediction model for individual progrnosis or diagnosis (TRIPOD): the TRIPOD Statement. BMC Med. 2015; 13(1): 1–10. doi: 10.1186/s12916-014-0241-z 25563062 PMC4284921

[22] Ministério da Saúde. Abordagem e Tratamento do Fumante - Consenso 2001. Rio de Janeiro: INCA, 2001. 38 p.

[23] Massago M, Dantas ML, Carolino IDR, Conegero CI. Uso de fármacos promove aumento na cessação do tabagismo. In: Cardoso NA, Rocha RR, editors. Ciência da Saúde. Ponta Grossa: Athena, 2019. pp. 129–135.

[24] Pearson’s correlation coefficient. In Kirch W, editors. Encyclopedia of Public Health. Dordrecht: Springer, 2008. pp. 1090–1091.

[25] Schratz P. oddsratio: Odds Ratio Calculation for GAM (M) s & GLM (M) s; 2022 [cited 2023 Jun 26]. Database. CRAN [Internet]. Available from: https://cran.r-project.org/web/packages/oddsratio/index.html.

[26] Gordon M. Introduction to forest plots, 2022 [cited 2023 Jun 26]. Database. CRAN [Internet]. Available from: https://cran.r-project.org/web/packages/forestplot/vignettes/forestplot.html.

[27] Kuhn M, Wing J, Weston S, Williams A, Keefer C, Engerlhardt A, et al. caret: Classification and Regression Training, 2023 [cited 2023 Jun 26]. Database. CRAN [Internet]. Available from: https://cran.r-project.org/web/packages/caret/index.html.

[28] Buuren S, Groothuis-Oudshoorn K, Vink G, Schouten R, Rbitzsch A, Rockenschaub P, et al. Package ‘Mice’, 2023 [cited 2023 Jun 26]. Database. CRAN. Available from: https://cran.r-project.org/web/packages/mice/mice.pdf.

[29] Coombes KR. Package ‘CrossValidate’, 2022 [cited 2023 Jun 26]. Database. CRAN. Available from: https://cran.r-project.org/web/packages/CrossValidate/CrossValidate.pdf.

[30] Ripley B. Package ‘tree’, 2023 [cited 2023 Jun 26]. Database. CRAN. Available from: https://cran.r-project.org/web/packages/tree/tree.pdf.

[31] Liaw A, Wiener M. randomForest: Breiman and Cutler’s Random Forests for Classification and Regression, 2022 [cited 2023 Jun 26]. Database. CRAN. Available from: https://cran.r-project.org/web/packages/randomForest/index.html.

[32] Yobero C. Determining credtworthiness for loan applications using C5.0 decision trees, 2018 [cited 2023 Jun 26]. Database. CRAN. Available from: https://rpubs.com/cyobero/C50.

[33] Friedman JH. Greedy function approximation: a Gradient Boosting Machine. Ann Stat. 2001; 29(5):1189–1232. doi: 10.1214/aos/1013203451

[34] Jiang W. Linear regression and logistic regression with missing covariates, 2021 [cited 2023 Jun 26]. Database. CRAN. Available from: https://cran.r-project.org/web/packages/misaem/vignettes/misaem.html.

[35] Pham T. K-Nearest Neighbors (KNN) – Using R, 2022. [cited 2023 Jun 26]. Database. CRAN. Available from: https://rpubs.com/pmtam/knn.

[36] Meyer D. Support vector machine [cited 2023 Jun 26]. Database: CRAN. Available from: https://cran.r-project.org/web/packages/e1071/vignettes/svmdoc.pdf.

[37] Chen T, He T, Benesty M, Khotilovich V, Tang Y, Cho H, et al. Extreme Gradient Boosting [cited 2023 Jun 26]. Available from: https://cran.r-project.org/web/packages/xgboost/index.html.

[38] Milborrow S. Package ‘rpart.plot’, 2022 [cited 2023 Jun 26]. Database. CRAN. Available from: https://cran.r-project.org/web/packages/rpart.plot/rpart.plot.pdf.

[39] Kharabsheh M, Meqdadi O, Alabed M, Veeranki S, Abbaddi A, Alzyound S. A machine learning approach for predicting nicotine dependence. Int J Adv Comput Sci Appl. 2019, 10(3): 179–184. doi: 10.14569/IJACSA.2019.0100323

[40] Zarikh R, Mathai A, Parikh S, Chandra Sekhar G, Thomas R. Understanding and using sensitivity, specificity and predictive values. Indian J Ophthalmol. 2008; 56(1):45–50. doi: 10.4103/0301-4738.37595 18158403 PMC2636062

[41] Patino CM, Ferreira JC. Understanding diagnostic tests. Part 2. J Bras Pneumol. 2017; 43(6):408. doi: 10.1590/S1806-37562017000000424 29340487 PMC5792038

[42] Probst P. Package ‘varImp”, 2022 [cited 2023 Jun 26]. Database. CRAN. Available from: https://cran.r-project.org/web/packages/varImp/varImp.pdf.

[43] Srisuphanunt M, Puttaruk P, Kooltheat N, Katzenmeier G, Wilairatan P. Prognostic indicators for the early prediction of severe dengue infection: a retrospective study in a University Hospital in Thailand. Trop Med Infect Dis. 2022, 7: 162–171. doi: 10.3390/tropicalmed7080162 36006254 PMC9416179

[44] Santos BS, Steiner MTA, Fenerich AT, Lima RHP. Data mining and machine learning techniques applied to public health problems: A bibliometric analysis from 2009 to 2018. Comput Ind Eng. 2019; 138; 106–120. doi: 10.1016/j.cie.2019.106120

[45] Minghui M, Chuanfeng Z. Application of Support Vector Machine to a small-sample prediction. Pet Explor Dev. 2015; 10(2) 72–75. doi: 10.3968/7830

[46] Schmidt A, Cappucciati M, Radua J, Rutiglaino G, Rocchetti M, Dell’Osso L, et al. Improving prognostic accuracy in subjects at clinical high risk for psychosis: systematic review of predictive models and meta-analysis sequential testing simulation. Schizophr Bull. 2017; 43(2): 375–388. doi: 10.1093/schbul/sbw098 27535081 PMC5605272

[47] Santos JDP, Duncan BB, Sirena SA, Vigo A, Abreu MNS. Indicadores de efetividade do Programa de Tratamento do Tabagismo no Sistema Único de Saúde em Minas Gerais, Brasil, 2008. Epidemiol. Serv. Saúde, Brasília. 2012; 21(4):579–588. doi: 10.5123/S1679-49742012000400007

[48] Planeta CS, Cruz FC. Bases neurofisiológicas da dependência do tabaco. Rev Psiquiatr Clin. 2005; 32(5): 251–258. doi: 10.1590/S0101-60832005000500002

[49] Kroon LA. Drug interactions with smoking. Am J Health Syst Pharm. 2007; 64(18): 1917–1921. doi: 10.2146/ajhp060414 17823102

[50] Schaffer SD, Yoon S, Zadazenski. A review of smoking cessation: potentially risk effects on prescribed medication. J Clin Nurs. 2009; 18(11): 1533–1540. doi: 10.1111/j.1365-2702.2008.02724.x 19490292

[51] França SAS, Neves ALF, Souza TAS, Martins NCN, Carneiro SR, et al. Factors associated with smoking cessation. Rev Saúde Pública. 2015; 49: 10–17. doi: 10.1590/s0034-8910.2015049004946 25741649 PMC4386556

[52] Arendartchuk D, Ayala ALM. Fatores associados à cessação do tabagismo entre participantes de um programa antitabagista em uma unidade básica de saúde de Joinville-SC. Rev APS. 2018; 21(4): 570–589. doi: 10.34019/1809-8363.2018.v21.16566

[53] Zhou X, Nonnemaker J, Sherrill B, Gilsenan AW, Coste F, West R. Attempts to quit smoking and relapse: factors associated with success or failure from the Attempt cohort study. Addict Behav. 2009; 34(4): 365–373. doi: 10.1016/j.addbeh.2008.11.013 19097706

[54] Chaiton M, Diemert L, Cohen JE, Bondy S, Selby P, Neri AP, et al. Estimating the number of quit attempts it takes to quit smoking successfully in a longitudinal cohort of smokers. BMJ Open. 2016; 6: e011045. doi: 10.1136/bmjopen-2016-011045 27288378 PMC4908897

[55] Wang R, Shenfan L, Song Y, Wang Q, Zhang R, Kuai L, et al. Smoking relapse reasons among current smokers with previous cessation experience in Shanghai: a cross-sectional study. Tob Induc Dis. 2023; 21:96–118. doi: 10.18332/tid/167963 37492763 PMC10364243

[56] Lee SE, Kim C, Im H, Jang M. Patterns and predictors of smoking relapse among inpatient smoking intervention participants: a 1-year follow-up study in Korea. Epidemiol Health. 2021; 43: e2021043. doi: 10.4178/epih.e2021043 34126705 PMC8298987

[57] Tohka J, Gils MV. Evaluation of machine learning algorithms for health and wellness applications: A tutorial. Comput Biol Med. 2021; 132: 104324–104338. doi: 10.1016/j.compbiomed.2021.104324 33774270

[58] Afzal Z, Schuemie M, Blijderveen JCV, Sen EF, Sturkenboom MCJM, Kors JA. Improving sensitivity for machine learning methods for automated case identification from free-text electronic medical records. BMC Med Inform Decis Mak. 2013; 13: 30–40. doi: 10.1186/1472-6947-13-30 23452306 PMC3602667

